# Sex-specific cardiac remodeling and metabolic reprogramming in IUGR F2 fetuses following glyphosate or glyphosate-based herbicide exposure

**DOI:** 10.3389/ftox.2026.1870011

**Published:** 2026-08-25

**Authors:** Ailín Almirón, Virginia Lorenz, Javier Adur, Juan Ignacio Etchart, Milena Durando, María Mercedes Milesi

**Affiliations:** 1 Instituto de Salud y Ambiente del Litoral (ISAL), Consejo Nacional de Investigaciones Científicas y Técnicas (CONICET) – Universidad Nacional del Litoral (UNL), Santa Fe, Argentina; 2 Cátedra de Fisiología Humana, Facultad de Bioquímica y Ciencias Biológicas (FBCB) – UNL, Santa Fe, Argentina; 3 Instituto de Investigación y Desarrollo en Bioingeniería y Bioinformática (IBB), CONICET – Universidad Nacional de Entre Ríos (UNER), Oro Verde, Entre Ríos, Argentina; 4 Laboratorio de Microscopía Aplicada a Estudios Moleculares y Celulares (LAMAE), Facultad de Ingeniería (FI) – UNER, Oro Verde, Entre Ríos, Argentina

**Keywords:** cardiac remodeling, glyphosate, glyphosate-based herbicides, intrauterine growth restriction, multigenerational effect

## Abstract

We previously demonstrated that perinatal exposure to a glyphosate-based herbicide (GBH) or pure glyphosate (Gly) induces intrauterine growth restriction (IUGR) in second-generation (F2) rats. Given the association between IUGR and fetal cardiac remodeling, we investigated whether ancestral exposure programs structural, mitochondrial, and metabolic alterations in F2 fetal hearts. Pregnant F0 rats received GBH or Gly (3.88 and 3.83 mg glyphosate/kg/day, respectively) from gestational day (GD) 9 to F1 weaning. F1 females were subsequently mated and euthanized at GD19 to collect male and female F2 fetal hearts. Both treatments increased relative heart weight in males, while GBH induced left ventricle dilation in both sexes, compatible with a globular phenotype. In males, Gly reduced cardiomyocyte proliferation and *Dio2* expression, and both treatments increased *Vcam1* expression. GBH also decreased mitochondrial DNA copy number while upregulating *Nd1* and mitochondrial biogenesis markers, suggesting a compensatory response. In females, GBH increased *Pdk4* and *Pgc1-α* expression, and Gly increased *Pparα* and *Acadl* expression, indicating a metabolic reprogramming. Autofluorescence lifetime imaging revealed sex- and treatment-dependent alterations in NAD(P)H and FAD lifetimes. Overall, effects were more pronounced in GBH-exposed lineage and in males, whereas Gly induced subtler alterations. These findings identify the fetal heart as a multigenerational target of herbicide exposure and highlight the contribution of co-formulants to developmental toxicity.

## 1 Introduction

Glyphosate-based herbicides (GBHs) are broad-spectrum agrochemicals, containing glyphosate (Gly) as the active ingredient. Over the past 2 decades, they have become the most used pesticides worldwide (Benbrook, 2016). Argentina, in particular, is currently the world’s third-largest consumer of pesticides, with GBHs accounting for more than 65% of the total volume applied (Aparicio et al., 2025), which amounts to 200 million liters annually (Montoya et al., 2023). Therefore, although Gly has a relatively short environmental half-life (Bento et al., 2019), its extensive and repeated application far exceeds its dissipation rate, making it a persistent pollutant in agricultural systems (Primost et al., 2017; Mac Loughlin et al., 2022). Its ubiquitous presence in several environmental matrices (Cheng et al., 2025) and biological samples (Gerona et al., 2022), has raised concerns regarding adverse outcomes in both human populations and non-target organisms (Klátyik et al., 2025).

Commercial GBHs are complex chemical mixtures that include not only Gly but also co-formulants that enhance herbicidal efficacy. These co-formulants have long been regarded as inert or toxicologically irrelevant compared with Gly itself. Consequently, regulatory safety assessments have primarily focused on the active ingredient, potentially underestimating the toxicity associated with exposure to the complete formulation (Klátyik et al., 2025). Nevertheless, accumulating experimental evidence indicates that the toxicity of GBHs may equal or even exceed that of Gly alone (Mesnage et al., 2019; Makame et al., 2023), highlighting the need to evaluate not only the isolated active principle, but also the biological effects of the full chemical mixture (Mesnage and Antoniou, 2018).

Human biomonitoring studies have demonstrated widespread exposure to Gly and have linked prenatal exposure to adverse birth outcomes, including reduced birthweight, preterm birth, and increased risk of neonatal intensive care unit admission (Silver et al., 2021; Gerona et al., 2022; Reynier and Rubin, 2025). Consistently, previous work from our laboratory have shown that GBH or Gly exposure during gestation and lactation causes adverse reproductive outcomes across two successive generation of rats, including implantation failure (Lorenz et al., 2020; Almirón et al., 2024), congenital anomalies (Milesi et al., 2018), intrauterine growth restriction (IUGR), impair postnatal development and increased neonatal mortality (Almirón et al., 2026). Furthermore, abnormal development was associated with oxidative imbalance in the intrauterine environment, and Gly was detected in amniotic fluid, providing evidence of its maternal–fetal transfer during critical windows of development (Almirón et al., 2026).

IUGR is a pathological condition in which the fetus fails to achieve its genetically determined growth potential. It represents one of the leading causes of neonatal morbidity and mortality and is strongly associated with an increased incidence of chronic diseases in adulthood, including cardiovascular disease, diabetes mellitus, and hypertension (Figueras and Gardosi, 2011). The fetal heart is particularly vulnerable to adverse intrauterine conditions, as cardiac development relies on tightly regulated processes of cellular growth, structural maturation and mitochondrial homeostasis (Botting et al., 2014). Disruption of these pathways during critical windows of development may result in cardiac and metabolic adaptive responses to growth-restricted conditions. Then, adaptative responses may progress to persistent changes in cardiac morphology and functional capacity, thereby increasing susceptibility to cardiovascular dysfunction later in life (Barker et al., 2006; Youssef et al., 2023; Wu et al., 2013). Given that cardiovascular disease remains the leading cause of mortality worldwide, identifying environmental exposures capable of disrupting fetal cardiac development is of major public health relevance.

Building on our previous findings that ancestral exposure of rats to Gly or a GBH induced IUGR and oxidative imbalance in the intrauterine environment of second-generation fetuses (Almirón et al., 2026), the present study aimed to investigate whether these alterations are associated with fetal cardiac remodeling and metabolic reprogramming. To address this, we analyzed cardiac histoarchitecture, mitochondrial homeostasis, and key molecular pathways involved in heart development and metabolism, focusing on identifying sex-dependent effects, thereby addressing the multigenerational and sexually dimorphic impact of Gly and GBH exposure.

## 2 Methodology

### 2.1 Animals

Inbred Wistar rats were housed at the Instituto de Salud y Ambiente del Litoral (ISAL, UNL-CONICET) under controlled environmental conditions (14-h light/10-h dark cycle) and with free access to food and water *(ad libitum)*. The experimental protocol was approved by the Ethical Committee of the Centro Científico Tecnológico of the Consejo Nacional de Investigaciones Científicas y Técnicas (CONICET), Santa Fe, Argentina (Case code: CEYSTE-CES 00710/2021). All procedures were conducted in accordance with the principles and procedures outlined in the Guide for the Care and Use of Laboratory Animals, published by the US National Academy of Sciences, and in compliance with the ARRIVE guidelines.

### 2.2 Chemicals

Glyphosate (N-(phosphonomethyl) glycine) (CAS Number: 1071-83-6), with a purity of 96%, was purchased from Sigma-Aldrich Inc. (Saint-Louis, MO, United States). The GBH formulation used in this study (Roundup Full II), is a water-soluble liquid formulation comprising 66.2% glyphosate potassium salt (equivalent to 54% w/v of glyphosate acid) as the active ingredient. The formulation also includes coadjuvants and inert ingredients not disclosed by the manufacturer.

### 2.3 Experimental design

Female rats in proestrus were caged overnight with males of proven fertility. The day that sperm was found in the vagina was designated as gestational day (GD) 1. Pregnant females (F0 generation) were randomly assigned to one of three groups (8 dams per group): Control, GBH or Gly. Dams were orally exposed from GD9 until weaning of their offspring (F1 generation) which occurred at postnatal day (PND) 21 (Milesi et al., 2018). Control group received laboratory pellet chow-based paste with vehicle (water), while GBH and Gly group received fed paste supplemented with either a commercial glyphosate-based formulation or pure glyphosate, at doses of 3.88 and 3.83 mg of glyphosate/kg body weight/day, respectively. These doses are in the order of magnitude of the reference dose (RfD) of 1 mg/kg body weight/day for glyphosate, as established by the Environmental Protection Agency (Perron et al., 2017) based on developmental toxicity studies. Their toxicological relevance is further supported by our previous findings using the same experimental design, where adverse reproductive outcomes were consistently observed, including implantation failure in F1 females (Lorenz et al., 2020) and IUGR in F2 fetuses (Almirón et al., 2026). Importantly, the internal exposure achieved in dams (0.0370 and 0.0130 mg/L for GBH and Gly groups, respectively) was comparable to serum concentrations reported in human biomonitoring studies, such as pregnant farmers (0.0175 mg/L; Kongtip et al., 2017) and non-occupationally exposed men (0.0141 mg/L; Filippi et al., 2023). Thus, the selected doses reproduce environmentally relevant exposures and yield internal concentrations consistent with those observed in humans, reinforcing their toxicological relevance.

**FIGURE 1:**
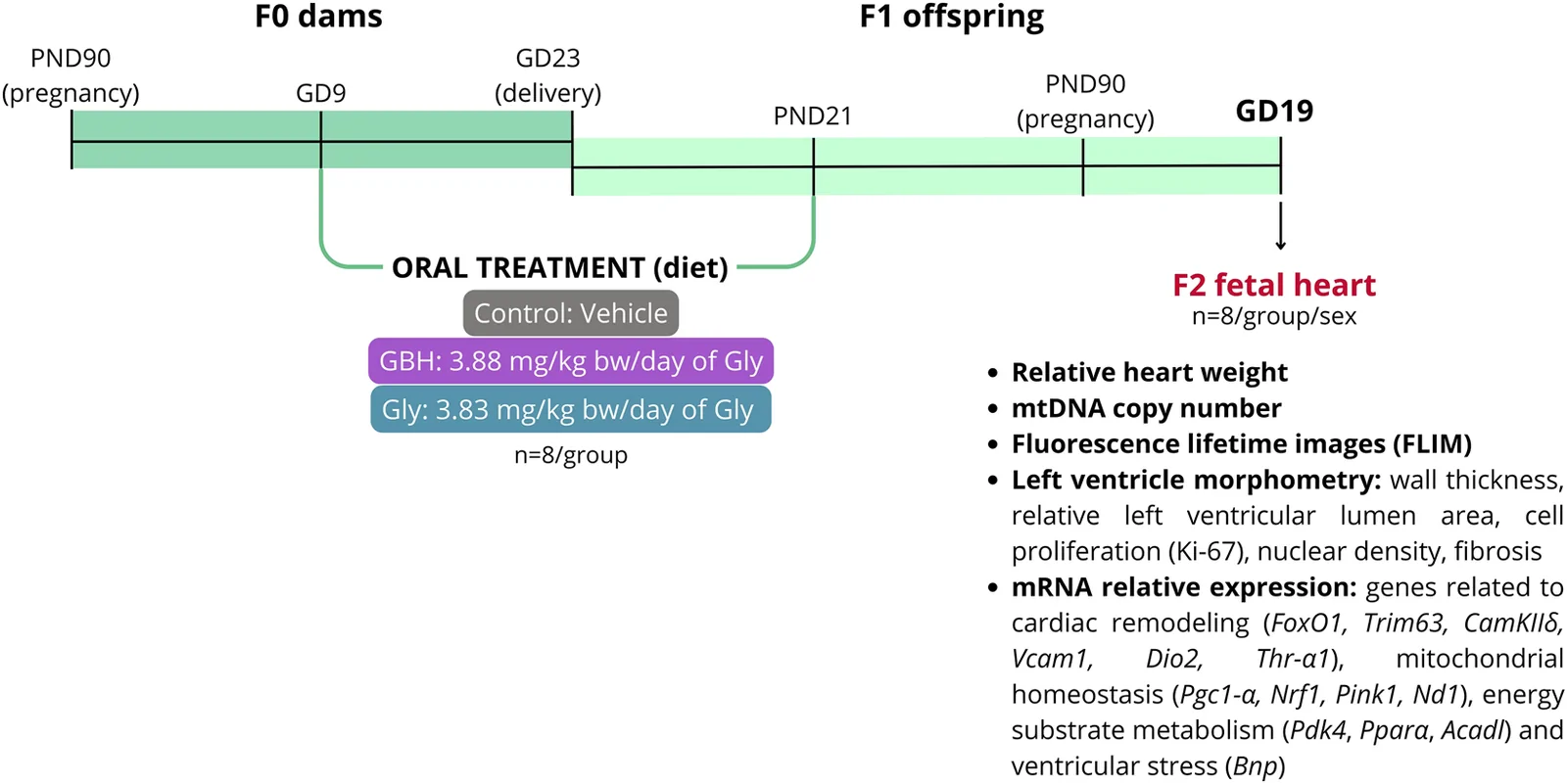
Schematic representation of the experimental protocol used to study the effects of perinatal exposure to GBH or Gly on F2 fetal heart development. PND: postnatal day; GD: gestational day.

Dams were allowed to deliver naturally and continue their respective treatments until PND21. Litters were standardized to eight pups per dam, maintaining a balanced sex ratio whenever possible. Sexually mature F1 females (n = 8/group, one per F0 dam) were then mated with unexposed proven fertility males and euthanized on GD19 using a pressurized CO_2_ chamber with a flow rate equivalent to 30% of the chamber volume/min and posterior decapitation according to the AVMA guidelines for the euthanasia of animals (Leary, 2020). Fetal and heart weight was recorded to estimate the relative heart weight. Placental tissue was also collected to determine fetal sex by real-time RT-qPCR amplification of the *Sry* gene (NM_012772.1) using the following primers: sense 5′-ATC​AGC​AAG​CAT​CTG​GGA-3′ and antisense 5′-TCT​CTC​TGT​GTA​GGG​TCT​TC-3′. Sex determination was performed according to Su and Loch-Caruso (2020), with minor modifications. Positive expression of the *Sry* gene confirmed that the samples were males. To minimize litter effects, a maximum of two male and two female fetuses per F1 dam were selected from each group. In each experimental group, the F2 fetuses were obtained from eight independent F1 dams, ensuring representation across multiple litters. From each litter, hearts from one male and one female were processed for histological analysis (n = 8 F2 fetuses/sex/experimental group), while another male and female heart were snap-frozen in liquid nitrogen and stored at −80 °C for subsequent RNA and DNA extraction (n = 8 F2 fetuses/sex/experimental group). A schematic representation of the experimental design is shown in Figure 1.

### 2.4 Histomorphological analysis

The histomorphological evaluation was performed on longitudinal heart sections (5 µm thick) stained with Mayer’s hematoxylin and eosin (H&E) and Masson’s trichrome under a light microscope (Olympus BH2 microscope; Olympus, Tokyo, Japan). Parameters were analyzed using FIJI software (ImageJ, National Institutes of Health, Bethesda, MD, United States, Schindelin et al., 2012). Left ventricular thickness was measured across the compact wall, from the endocardial to the epicardial surface. The relative left ventricular lumen area was estimated as the ratio between the total left ventricular area and the area occupied by the lumen. Nuclear density was determined by counting the total nuclei within the compact wall of the left ventricle and expressed as nuclei/µm^2^. All measurements were performed in ten sections per sample separated 25 µm from each other.

### 2.5 Immunohistochemistry assay

Immunohistochemistry assay was performed to determine left ventricular proliferation by assessing the Ki-67 marker, following protocols previously described (Almirón et al., 2024). Briefly, heart longitudinal sections (5 µm thick) were deparaffinized, rehydrated in graded ethanol, and subjected to microwave antigen retrieval. Endogenous peroxidase activity and non-specific binding sites were blocked before overnight incubation at 4 °C with the anti-Ki-67 primary antibody (1:1600, Gomez et al., 2020), following a 30-min incubation with biotinylated secondary anti-mouse antibody (1:200, Sigma-Aldrich Inc., St. Louis, MO, United States). Reactions were developed using the avidin-biotin-peroxidase method with diaminobenzidine (Sigma-Aldrich) as a chromogen substrate. Each immunohistochemical run included positive and negative controls; no background signal was detected in negatives. Samples were counterstained with Mayer’s hematoxylin (Biopur, Rosario, Argentina). Proliferation index was calculated using a point grid as the volume fraction (Vv = Pi/P) of Ki-67 positive cells (Weibel, 1969).

### 2.6 DNA and RNA extraction and retrotranscription

Samples of heart were individually homogenized in TRIzol (Invitrogen, Carlsbad, CA, United States), and DNA and RNA were extracted according to the manufacturer’s instructions. Total DNA and RNA concentration was assessed by A260, and the samples were stored at −80 °C until used. For retrotranscripcion, 1 μg of total RNA was reverse transcribed into cDNA as reported in our previous work (Lorenz et al., 2019).

### 2.7 Real-time quantitative PCR (RT-PCR)

A standardized RT-qPCR protocol was applied to evaluate the mRNA relative expression of genes involved in cardiac remodeling (*FoxO1* and their major downstream effectors *Vcam1, Dio2, CamKII-δ,* and *Trim63*), thyroid hormone receptor α1 (*Thr-α1*), mitochondrial homeostasis (*Pgc1-α, Nrf1, Pink1* and *Nd*-1), energy substrate metabolism (*Pdk4, Pparα, Acadl*) and ventricular stress (*Bnp,* used as biomarker of left ventricle injury). Ribosomal protein L19 (*Rpl19*) was selected as the housekeeping gene (Lorenz et al., 2019). Primer sequences were designed using the NCBI primer-blast tool and are listed in Table 1 mRNA levels were quantified using the Real-Time DNA Step One Cycler (Applied Biosystems Inc., Foster City, CA, United States) based on the cycle threshold (Ct) method (Higuchi et al., 1993). The results for each target were expressed as fold change relative to control values using the standard curve method (Čikoš et al., 2007). The target gene quantities were derived from the standard curve, normalized to *Rpl19*, and compared to control sample. Ct values for *Rpl19* were not different between experimental groups.

**TABLE 1:** Primer sequences and PCR products for real-time RT-PCR analysis.

Target gene (Refsec ID)	Primer sense (5’-3’)	Primer antisense (5’-3’)	Product size (bp)	Annealing temperature (°C)
*Rpl19* (NM_031103.1)	AGC​CTG​TGA​CTG​TCC​ATT​CC	TGG​CAG​TAC​CCT​TCC​TCT​TC	99	60
*FoxO1* (NM_001191846.3)	TCT​ACG​AGT​GGA​TGG​TGA​AGA​GTG	AGG​GAC​AGA​TTG​TGG​CGA​A	100	55
*Dio2* (NM_031720.5)	GCGACCTGACCACCTTTT	ACC​AAC​AGG​AAG​TCA​GCC​AC	86	55
*Thr-α1* (NM_001017960.2)	CTGGAGGTCTTTGAGGAT	GTATCTATCCAAGGACGG	153	55
*CamKII-δ* (NM_012519.2)	CCG​TGA​TGG​GAA​GTG​GCA​GAA	GGA​ATA​CAG​GGT​GGC​TTG​ATG​GG	78	56
*Trim63* (NM_080903.2)	GAC​TTT​GGG​ACA​GAT​GAG​GAG​G	TTC​ATT​GGT​GTC​CCT​CTG​TGG	91	55
*Vcam1* (NM_012889.2)	GGT​GTT​GAG​GAT​GAG​CAC​T	GCA​GTT​GAC​AGT​GAC​AGG​T	156	54
*β-actin* (NC_086030.1)	GGC​ACC​ACA​CTT​TCT​ACA​ATG​AGC	ATT​GAG​AAA​GGG​CGT​GGC​TGA	171	61
*Nd-1* (NC_001665.2:2740-3694)	TCA​TAA​AAG​AAC​CCA​TAC​GCC​CC	AAG​GGG​GTG​AGG​TAT​TGG​TAA​GG	119	55
*Pgc1-α* (NM_031347.1)	GCC​ACT​ACA​GAC​ACC​GCA​CAC​AT	CTC​TCT​GCG​GTA​TTC​GTC​CCT​CT	143	58
*nrf1* (NM_001100708.1)	TGG​CAA​CAG​GGA​AGA​AAC​GG	TGC​TGT​CCC​ACT​CGT​GTC​GTA​T	133	56
*Pink1* (NM_001438119.1)	CAA​GTC​GGT​GCC​TCC​AGA​AA	TAG​AAG​ATG​CTC​ACC​CCA​GAG​G	127	59
*Pdk4* (NM_053551.2)	GGA​TTA​CTG​ACC​GCC​TCT​TTA​G	GCA​AGC​CGT​AAC​CAA​AAC​C	102	55
*Pparα* (NM_013196.2)	CGG​GTC​ATA​CTC​GCA​GGA​AA	GCA​GCA​GTG​GAA​GAA​TCG​GA	153	58
*Acadl* (NM_012819.3)	CAT​CGG​TGG​GGA​CTT​GTT​GT	TCT​GGG​GGA​TAA​ACT​GCT​CG	155	56
*Bnp* (NM_031545.1)	CCA​GTC​TCC​AGA​ACA​ATC​CAC	CGG​TCT​ATC​TTC​TGC​CCA​AA	219	56

*Rpl19*, ribosomal protein L19; *FoxO1*, forkhead box O1; *Dio2*, iodothyronine deiodinase 2; *Thr-α1*, thyroid hormone receptor alpha 1; *CamKII- δ,* calcium/calmodulin-dependent protein kinase II, delta; *Trim63*, tripartite motif containing 63; *Vcam1*, vascular cell adhesion molecule 1; β-*actin*, beta-actin; *Nd1*, NADH, dehydrogenase subunit 1 (mitochondrially encoded); *Pgc1*-α, peroxisome proliferator-activated receptor gamma coactivator 1 alpha; *Nrf1*, nuclear respiratory factor 1; *Pink1*, PTEN-induced kinase 1; *Pdk4*, pyruvate dehydrogenase kinase isozyme 4; *Pparα,* peroxisome proliferator activated receptor alpha; *Acadl*, acyl-CoA, dehydrogenase, long chain; *Bnp,* B-type natriuretic peptide.

### 2.8 Mitochondrial DNA copy number

Total DNA was used to determine mitochondrial DNA (mtDNA) copy number by qPCR using the Real-Time DNA Step One Cycler (Applied Biosystems, Foster City, CA, United States). The mitochondrial gene *Mt-Nd1* was used as the target gene, and *β-actin* was used as the nuclear reference gene for normalization.

Relative mtDNA copy number was calculated using the comparative Ct (2^-ΔΔCt^) method, as reported by Bryant et al. (2022). For each sample, ΔCt value was obtained by subtracting the Ct of the nuclear gene (*β-actin*) from the Ct of the mitochondrial gene (*Mt-Nd1*), thereby normalizing mitochondrial to nuclear DNA content. The ΔΔCt was calculated relative to the control group, and results were expressed as relative mtDNA copy number. Primer sequences are listed in Table 1.

### 2.9 Fluorescence lifetime imaging microscopy

Autofluorescence lifetime images (AF-FLIM) analysis was applied to evaluate functional insight into metabolic organization in fetal cardiac tissue. NADH, NADPH and FAD are key cofactors whose fluorescence lifetimes reflect binding states and provide information on mitochondrial activity (Datta et al., 2020). During oxidative phosphorylation, NADH autofluorescence decreases upon oxidization to nonfluorescent NAD + at complex I, whereas FAD autofluorescence increases when FADH_2_ is oxidized at complex II (Tan and Dunning, 2022). The fluorescence of NADH and NADPH are difficult to distinguish, and their combined fluorescence is referred to as NAD(P)H. For NAD(P)H, shorter lifetimes correspond to the free form and longer lifetimes to protein-bound species. In contrast, protein-bound FAD exhibits shorter lifetimes, whereas free FAD shows longer lifetimes (Bird et al., 2005; Skala et al., 2010). This approach enables sensitive detection of bioenergetics alterations independent of transcriptional changes, thereby revealing early metabolic vulnerabilities.

The FLIM images were acquired by a time-correlated single-photon counting system (SPC-150, Becker and Hickl, Berlin, Germany) on a laser scanning confocal microscope (LSM 880- Carl Zeiss AG, Germany) with a Plan-Apochromat 63X, NA 1.4 oil-immersion objective, providing high-resolution imaging for accurate visualization of left ventricular cells. The samples were excited by a 405 nm ps laser (50 MHz, BDL-405-SMC, Becker and Hickl, Berlin, Germany). A filter cube with a long-pass beam splitter (LP495 nm at 45°) separated the emission into two channels: one arm with a 420–480 nm bandpass filter (NAD(P)H channel) and the other with a 500–550 nm bandpass filter (FAD channel). Each image (512 × 512 pixels) was acquired over 120s and detected using an external GaAsP PMT detector (BIG2). At least three different regions of interest (ROI) were imaged for each sample. In the selection of the ROIs, special care was taken to not capture the signal from the red blood cells.

AF-FLIM images were fitted with double-exponential decay models using the commercial SPCImage software (SPCImage v.8.8, Becker and Hickl, Berlin, Germany). Fits with χ^2^_r_ < 1.3 were considered acceptable, taking into account photon statistics and the intrinsic variability of autofluorescence signals. The lifetime matrix was calculated, and lifetime spectra was exported to Microsoft Excel for further analysis. The mean lifetime of each pixel τ_m_ can be obtained by the following formula: τ_m_ = a_1_τ_1_ + a_2_τ _2_. Therefore, for channel 1, a_1_ and a_2_ represent the relative contributions of free and protein-bound NAD(P)H, and τ_1_ and τ_2_ their respective fluorescence lifetimes. For channel 2, a_1_ and a_2_ represent the relative contributions of protein-bound and free FAD, and τ_1_ and τ_2_ their respective fluorescence lifetimes, all determined at each pixel.

### 2.10 Statistical analysis

Statistical analyses were performed using GraphPad Prism version 5.03 (GraphPad Software, San Diego, CA, United States). Data normality was assessed using the Shapiro–Wilk test, and homogeneity of variances was evaluated with Levene’s test. When assumptions were met, one-way ANOVA was performed, and significant effects were further explored followed by Tukey’s *post hoc* test for multiple comparisons. For datasets that did not meet the assumptions of normality and/or homogeneity of variance, the non-parametric Kruskal–Wallis was applied, and significant effects were further explored using Dunn’s test for multiple-comparison. To compare male and female data, two-way ANOVA analysis was performed, followed by Fisher’s LSD test. Results are expressed as mean ± SEM. A p-value<0.05 was considered statistically significant.

## 3 Results

### 3.1 GBH and Gly exposure increased relative heart weight in F2 male fetuses

**FIGURE 2:**
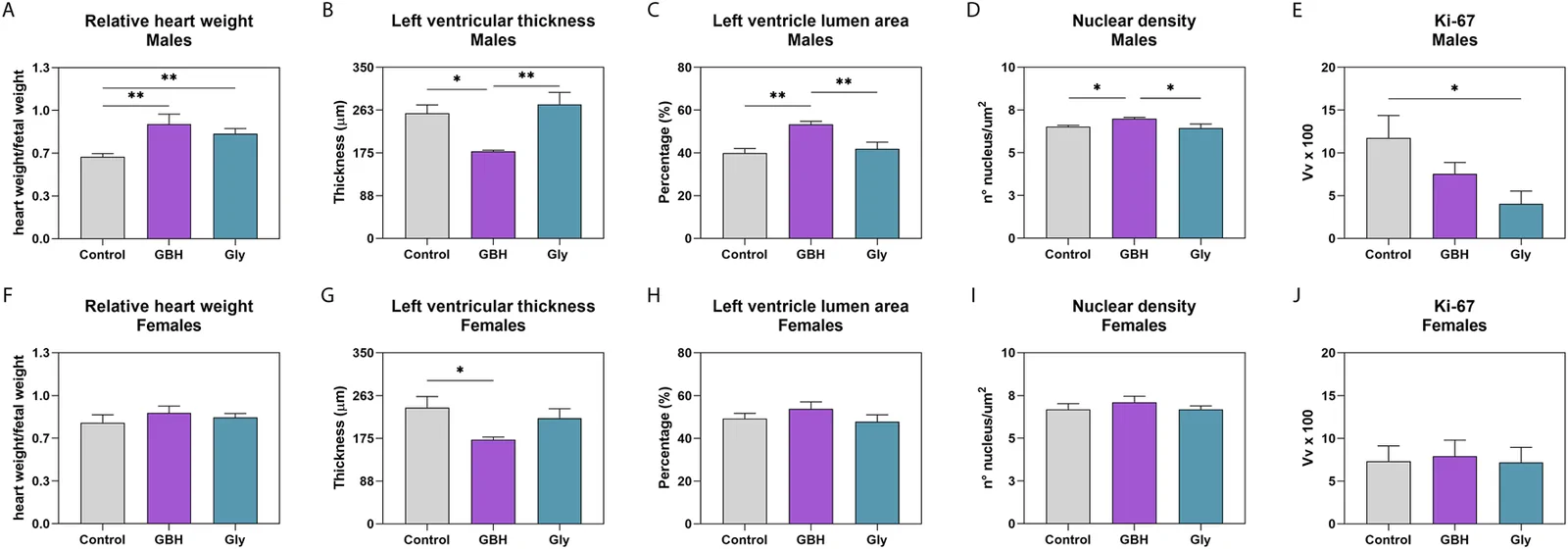
Effects of GBH or Gly exposure on fetal cardiac histomorphology and proliferation in F2 male **(A–E)** and female fetuses **(F–J)**. **(A,F)** Relative heart weight normalized to fetal body weight. **(B,G)** Left ventricular wall thickness. **(C,H)** Left ventricular lumen area expressed as a percentage of total ventricular area. **(D,I)** Nuclear density in the left ventricular myocardium. **(E,J)** Cardiomyocyte proliferative activity determined by Ki-67–positive nuclei. Data are presented as mean ± SEM. Statistical differences are indicated by asterisks (*p < 0.05, **p < 0.01).

As shown in Table 2, fetal body weight was significantly reduced in both F2 males and females exposed to GBH or Gly compared with their Control counterparts (p < 0.01). Although absolute heart weight remained unchanged across groups, relative heart weight was significantly increased in F2 males exposed to GBH or Gly compared with Control males (p < 0.01, Figure 2A). In contrast, no differences were observed among female fetuses (Figure 2F). Notably, Control females showed higher relative heart weight than Control males (p < 0.05, Table 2).

**TABLE 2:** Fetal body and heart weights of F2 male and female fetuses.

Analyzed parameters	Control	GBH	Gly
*Fetal body weight (mg)*			
Male	1285 ± 10.88^a^	1000 ± 40.52^b^	1064 ± 29.75^b^
Female	1244 ± 10.72^a^	1095 ± 12.22^b^	1111 ± 9.59^b^
*Fetal heart weight (mg)*			
Male	7.988 ± 0.31	8.586 ± 0.61	8.463 ± 0.31
Female	10.100 ± 0.69	9.217 ± 0.48	8.983 ± 0.30
*Relative heart weight*			
Male	0.622 ± 0.02^a#^	0.871 ± 0.08^b^	0.800 ± 0.04^b^
Female	0.769 ± 0.06	0.843 ± 0.05	0.809 ± 0.03

Data are presented as mean ± SEM. Different superscript letters (a, b) indicate statistically significant differences (p < 0.05) between groups of the same sex; # denotes significant differences between males and females of the same group.

### 3.2 GBH and Gly exposure differentially affected cardiac histomorphology and cardiomyocyte proliferation in a sex-dependent manner

**FIGURE 3:**
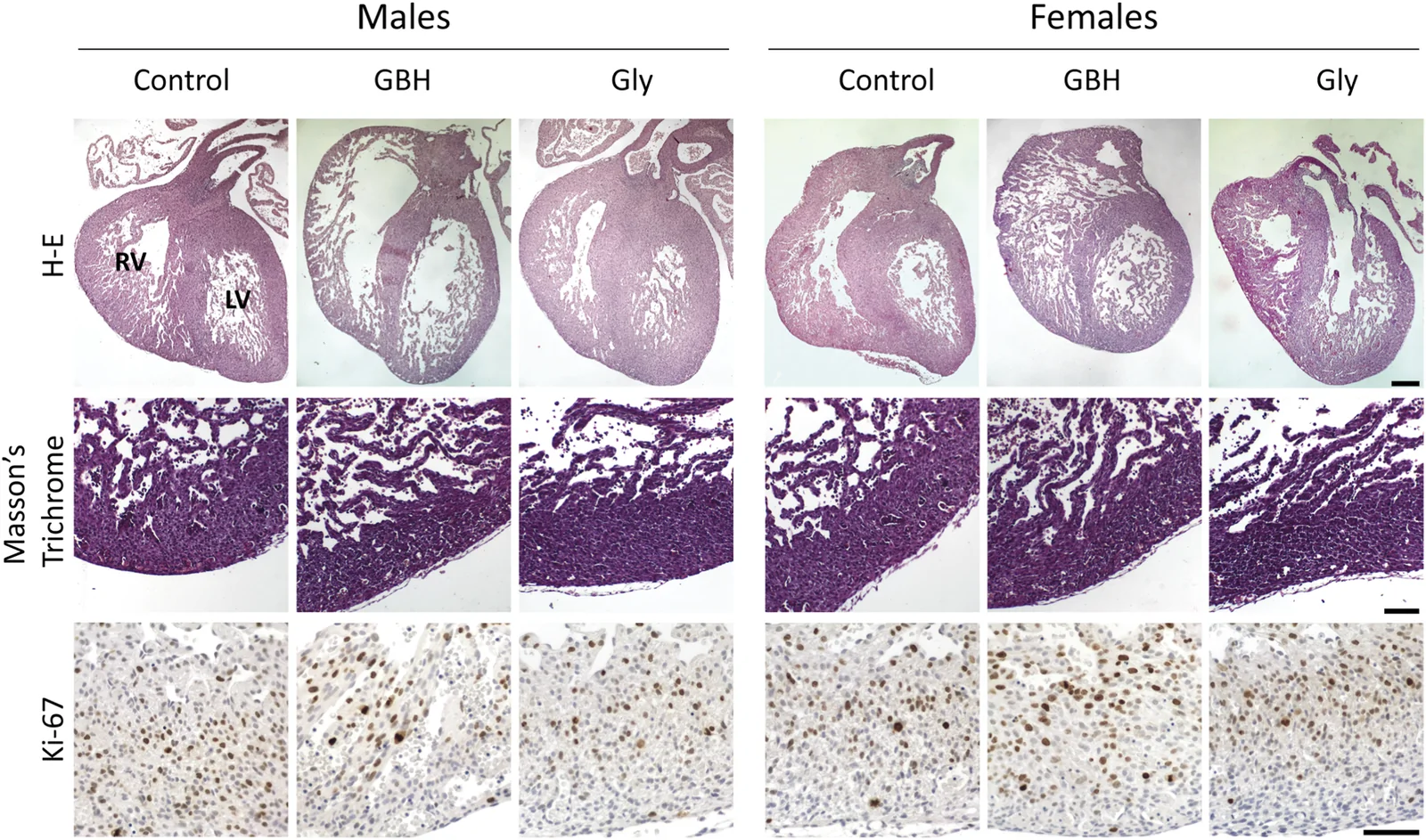
Effects of GBH or Gly exposure on fetal cardiac morphology, proliferation, and fibrosis in F2 male (left panels) and female (right panels) fetuses. Top row: Hematoxylin–eosin (H&E) staining at low magnification showing overall cardiac morphology. RV: right ventricle, LV: left ventricle (scale bar: 500 μm). Second row: Masson’s trichrome staining showing myocardial tissue architecture and the absence of interstitial fibrosis across experimental groups. Third row: Immunohistochemical detection of Ki-67–positive nuclei (brown staining), indicating cardiomyocyte proliferative activity. Images are representative of each experimental group. Scale bars for higher magnification images: 50 μm.

In male fetuses, left ventricular wall thickness was reduced in the GBH group compared with both Control (p < 0.05) and Gly (p < 0.01), accompanied by an increase in lumen area compatible with a more globular cardiac shape (p < 0.01, Figures 2B,C). In female fetuses, left ventricular wall thickness was also reduced in the GBH group compared with Control (p < 0.05; Figure 2G), although no differences in lumen area were observed among groups (Figure 2H). No interstitial fibrosis was detected by Masson’s trichrome staining in cardiac tissue from Control, GBH-, or Gly-exposed fetuses of either sex. Representative histological images stained with H&E and Masson’s trichrome are shown in Figure 3.

Cardiomyocyte proliferation in the left ventricular wall was significantly reduced in F2 male fetuses exposed to Gly compared with Controls (p < 0.05), whereas no differences were detected in GBH-exposed males (Figure 2E). Conversely, nuclear density in the left ventricle was significantly increased in GBH-exposed males compared with both Control and Gly (p < 0.05; Figure 2D). In female fetuses, neither cardiomyocyte proliferation nor nuclear density differed among experimental groups (Figures 2I,J). Representative images illustrating these results are shown in Figure 3.

The comparison between males and females revealed reduced left ventricular thickness in females from the Gly group compared to their male counterparts (p < 0.05, Supplementary Figure S1B), while females in the Control group had an increased left ventricular lumen area compared to males (p < 0.05, Supplementary Figure 1C). No differences in nuclear density or cardiomyocyte proliferation were observed between sexes (Supplementary Figures 1D,E, respectively).

### 3.3 GBH and Gly modulate key molecular pathways involved in cardiac remodeling in a sex-dependent manner

**FIGURE 4:**
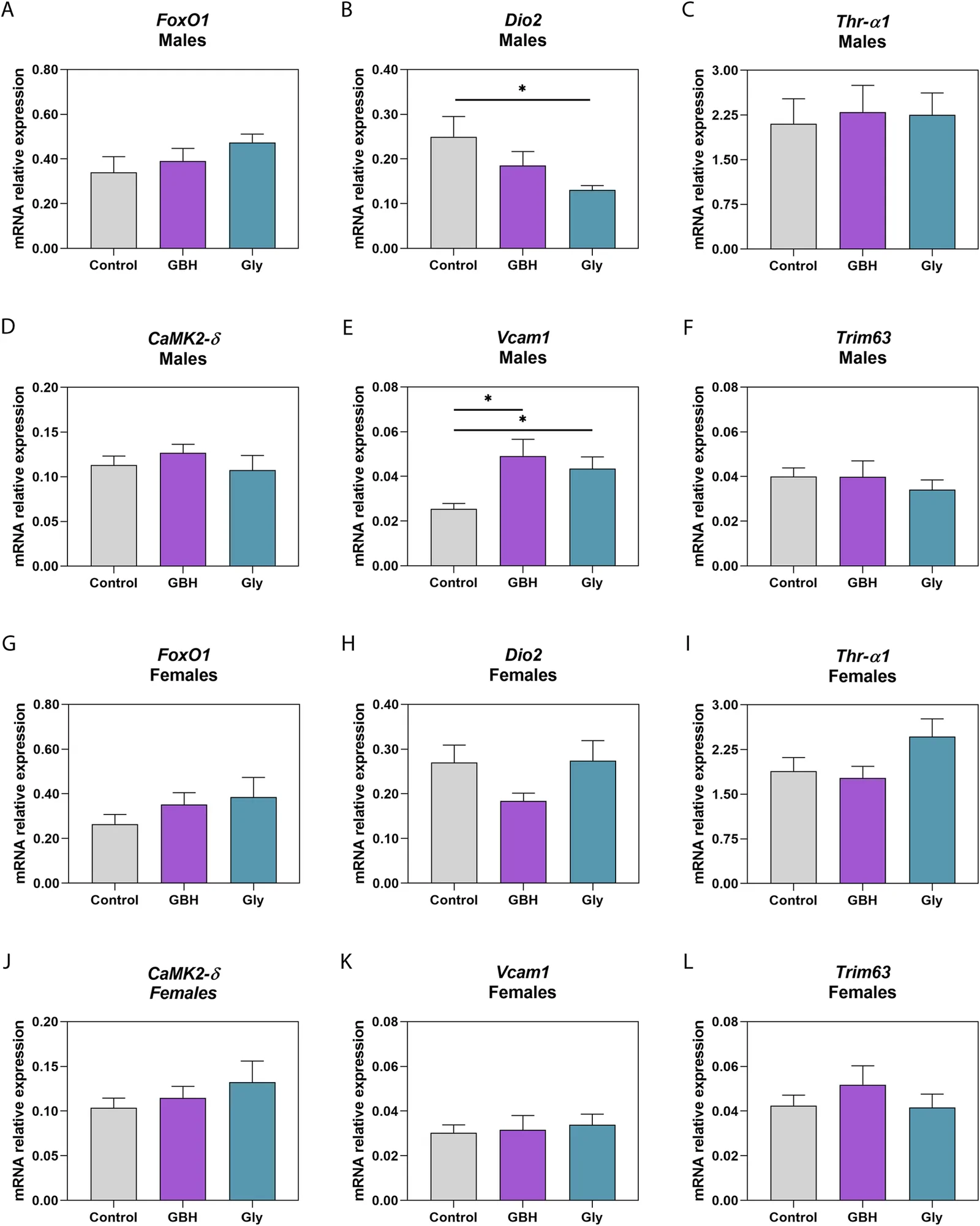
Effects of GBH or Gly exposure on genes involved in cardiac remodeling in fetal cardiac tissue of F2 male **(A–F)** and female **(G–L)** fetuses. Relative mRNA expression levels of **(A,G)** *FoxO1*, **(B,H)** *Dio2*, **(C,I)** *Thr-α1*, **(D,J)** *Camk2δ*, **(E,K)** *Vcam1*, and (F, L) *Trim63*. Data are presented as mean ± SEM. Statistical significance is indicated by asterisks (*p < 0.05).

In male fetuses, no significant differences were observed in *FoxO1, Thr-α1*, *Camk2δ,* and *Trim63* gene expression (Figures 4A,C,D,F, respectively). However, *Dio2* mRNA levels were significantly reduced in the Gly group compared with Control (p < 0.05; Figure 4B), whereas *Vcam1* expression was markedly increased in both GBH and Gly groups relative to Control (p < 0.05; Figure 4E). In female fetuses, no significant differences were observed in *FoxO1*, *Camk2δ*, *Trim63*, *Vcam1*, *Dio2* and *Thr-α1* gene expression (Figures 4G–L).

Comparison between sexes showed that *Dio2* mRNA levels were reduced in Gly males compared to Gly females (p < 0.05, Supplementary Figure 2B). Moreover, *Vcam1* mRNA levels were lower in GBH females than in GBH males (p < 0.05, Supplementary Figure 2D). No differences were detected in *FoxO1*, *Thr-α1, Camk2δ*, and *Trim63* mRNA levels when males were compared to females (Supplementary Figures S1A, C, E, F).

### 3.4 GBH exposure reduces mtDNA copy number and modulates mitochondrial homeostasis in a sex-dependent manner in F2 hearts

**FIGURE 5:**
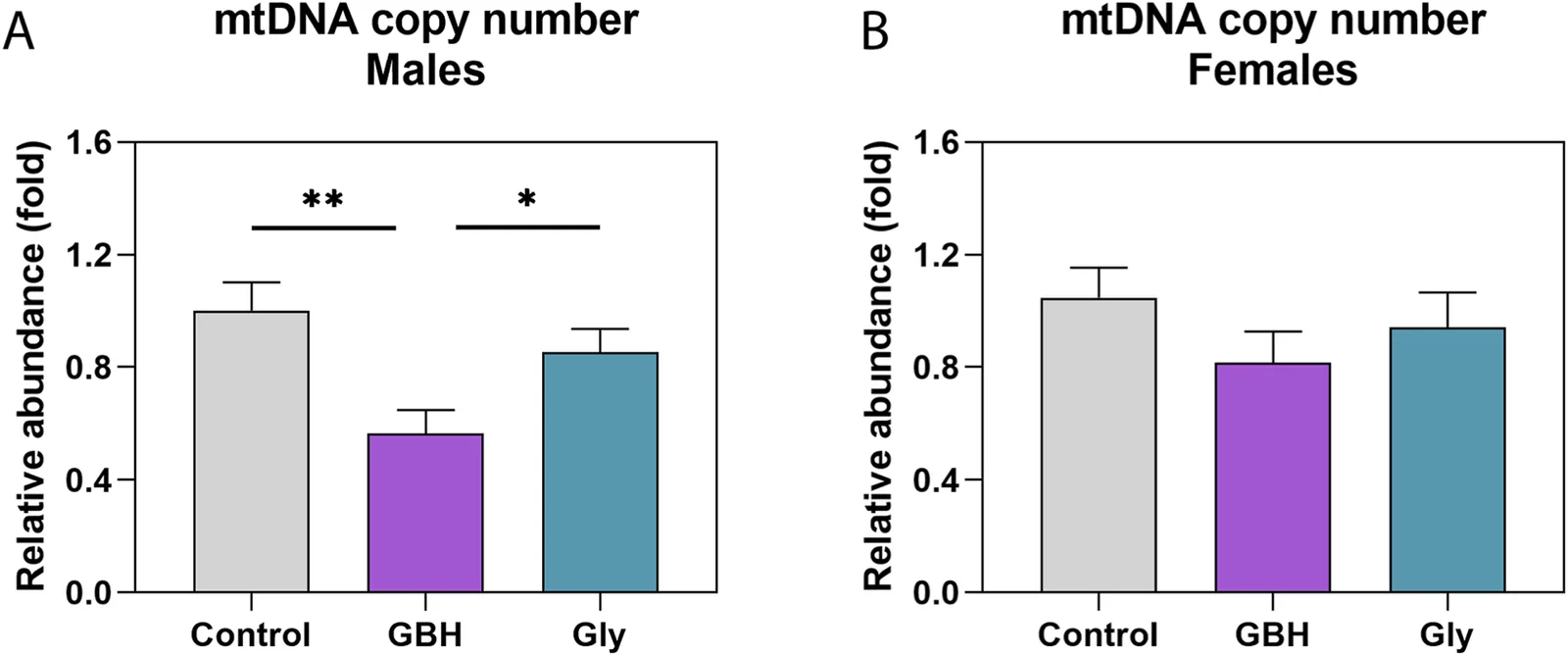
Effects of GBH or Gly exposure on relative mitochondrial DNA (mtDNA) copy number in fetal cardiac tissue of F2 male **(A)** and female **(B)** fetuses. Data are presented as mean ± SEM. Statistical differences are indicated by asterisks (*p < 0.05, **p < 0.01).

**FIGURE 6:**
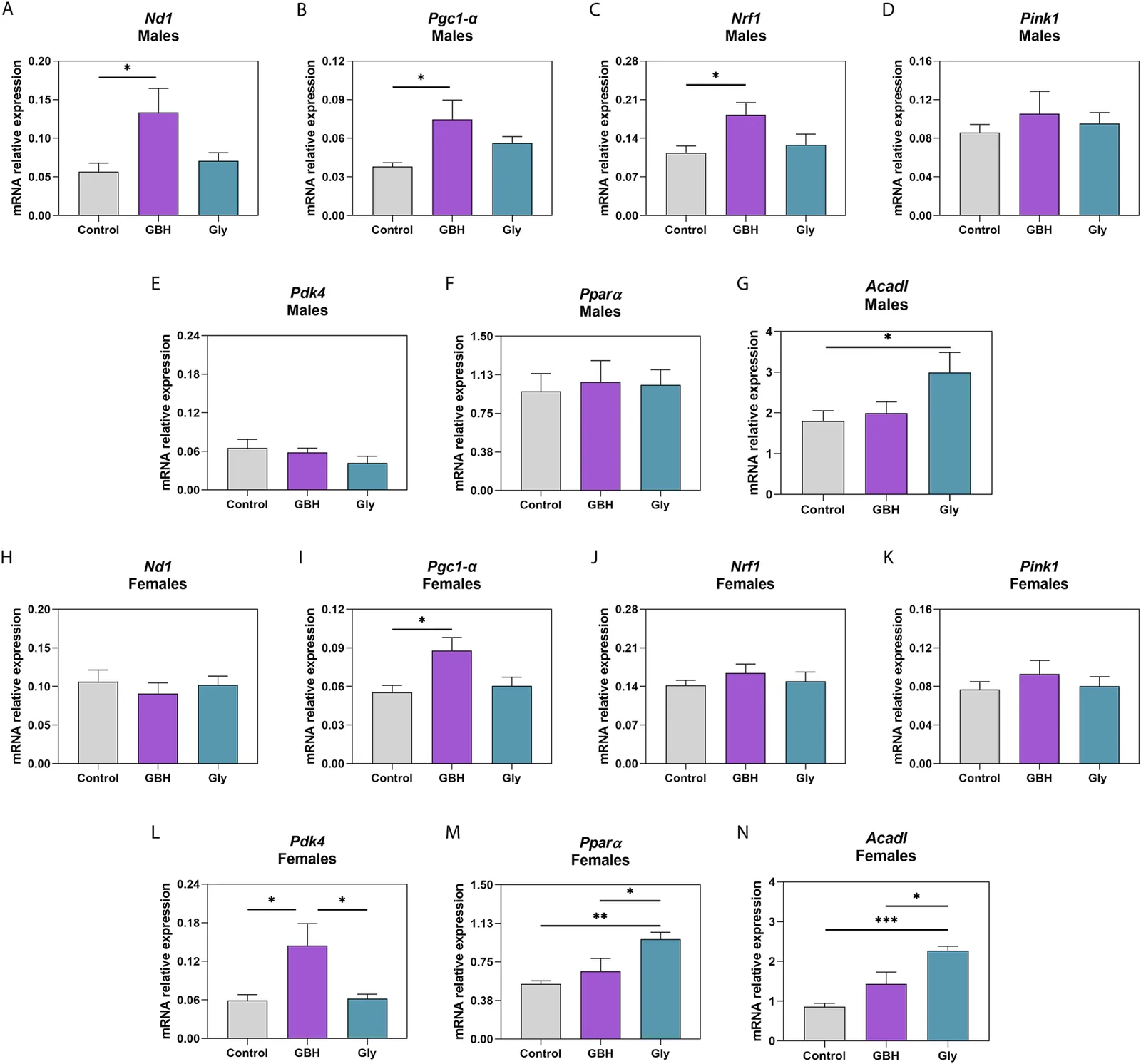
Effects of GBH or Gly exposure on mitochondrial regulatory pathways in fetal cardiac tissue of F2 male **(A–G)** and female fetuses **(H–N)** Relative mRNA expression levels of **(A,H)** *Nd1*, **(B,I)** *Pgc1α*, **(C,J)** *Nrf1*, **(D,K)** *Pink1,* **(E,L)** *Pdk4*, **(F,M)** *Pparα*, and **(G,N)** *Acadl*. Data are presented as mean ± SEM. Statistical differences are indicated by asterisks (*p < 0.05).

In male fetuses, mtDNA copy number was significantly decreased in the GBH group compared with both Control (p < 0.01) and Gly males (p < 0.05) (Figure 5A). However, *Nd1* was upregulated in GBH-exposed males relative to Control (p < 0.05; Figure 6A). Genes involved in mitochondrial biogenesis were also increased, with elevated *Pgc1-α* (p < 0.05; Figure 6B) and *Nrf1* expression (p < 0.05; Figure 6C) in GBH-exposed males respect to Control males. In contrast, expression of the mitophagy-related gene *Pink1* did not differ among groups (Figure 6D). Regarding genes involved in energy substrate metabolism, the negative regulator of glucose oxidation, *Pdk4*, and the inducer fatty acid oxidation, *Pparα,* did not differ among males (Figures 6E,F). Nonetheless, *Acadl* expression, which encodes the first enzyme of long-chain fatty acid β-oxidation in the mitochondria, was increased in Gly males respect to Controls (p < 0.05; Figure 6G).

In female fetuses, mtDNA copy number was comparable among experimental groups (Figure 5B). However, *Pgc1-α* and *Pdk4* expression was significantly increased in the GBH-exposed females compared with Control (p < 0.05; Figures 6I,L respectively), and *Pparα* and *Acadl* expression was increased in Gly-exposed females compared with both Control and GBH females (p < 0.01 vs. Control, p < 0.05 vs. GBH; Figures 6M,N respectively). Finally, *Nd1*, *Nrf1*, and *Pink1* levels showed no differences among females (Figures 6H,J,K respectively).

Comparison between females and males showed that *Nd1* mRNA levels were increased in female Control group compared to male Controls (p < 0.05, Supplementary Figure 3B). Likewise, increased *Pdk4* mRNA levels were detected in GBH females in relation to male counterparts (p < 0.001, Supplementary Figure 3F). Control females exhibited lower *Pparα* and *Acadl* expression than their males counterparts (p < 0.05, Supplementary Figure 3G, H, respectively), and GBH females showed lower *Pparα* expression than GBH males (p < 0.05, Supplementary Figure 3G). Finally, the mtDNA copy number and the expression of *Pink1, Pgc1-α* and *Nrf1* showed no changes between males and females (Supplementary Figures 3A, C, D, E).

### 3.5 Sex-dependent changes in NAD(P)H and FAD autofluorescence lifetimes in fetal cardiac tissue

**FIGURE 7:**
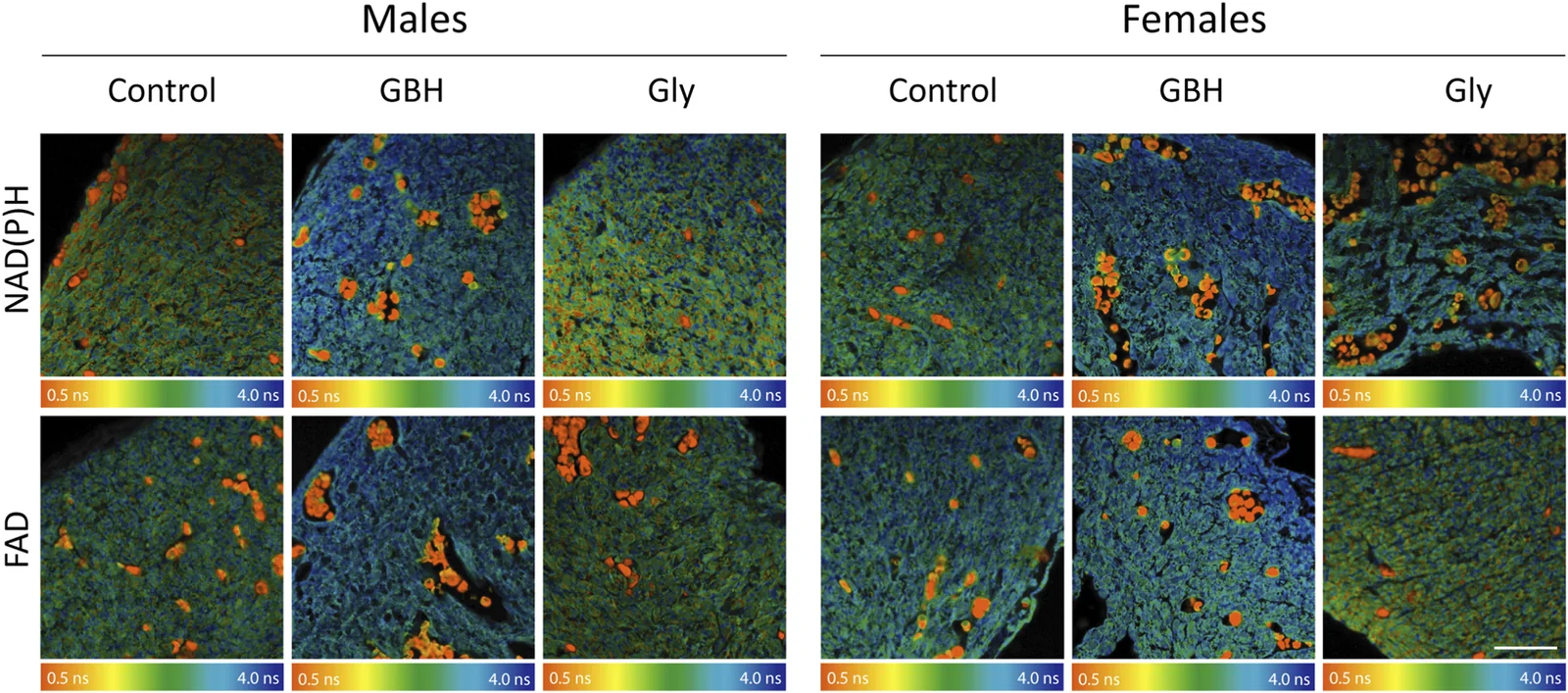
Effects of GBH or Gly exposure on fluorescence lifetime properties of NAD(P)H and FAD in fetal cardiac tissue. Representative autofluorescence lifetime imaging (AF-FLIM) images of NAD(P)H (top panels) and FAD (bottom panels) acquired in the left ventricle of F2 male (left) and female (right) fetuses from Control, GBH, and Gly groups. The color scale represents fluorescence lifetime values (ns), with identical scaling applied across all groups to allow direct visual comparison. Images illustrate the spatial distribution of fluorescence lifetimes. Lifetime components correspond to free and protein-bound NAD(P)H (τ1 and τ2, respectively) and protein-bound and free FAD (τ1 and τ2, respectively). Regions of interest were selected to exclude blood cells and to focus on myocardial tissue. Scale bar: 50 µm.

Table 3 and Figure 7 summarize the fluorescence lifetime parameters obtained by AF-FLIM for NAD(P)H and FAD in fixed fetal cardiac tissue. In Gly-exposed males, the mean lifetime (τm) and both short (τ1) and long (τ2) components of NAD(P)H were significantly reduced compared to Controls (p < 0.05), while the relative fractions α1 and α2 remained unchanged. In contrast, GBH-exposed males showed a significant decrease in FAD τm compared with Controls (p < 0.05). In females, a distinct pattern was observed. Gly exposure led to a decrease in the relative fraction of free NAD(P)H (α1) and a corresponding increase in the protein-bound fraction (α2) (p < 0.05), together with an increase in the τ1 component of FAD (p < 0.05). No significant changes in NAD(P)H or FAD lifetime parameters were observed in GBH-exposed females compared to Controls. It is important to mention that although chemical fixation can influence absolute fluorescence lifetimes (Berezin and Achilefu, 2010; Datta et al., 2020), AF-FLIM provided robust comparative data across groups in this study. Therefore, the observed differences reflect treatment- and sex-dependent alterations in cofactor organization and microenvironment.

**TABLE 3:** FLIM parameters from F2 hearts.

Excitation/pulsated laser (nm)	Channel/emission (nm)	τ_m_ (ns)	τ_1_ (ns)	α_1_ (%)	τ_2_ (ns)	α_2_ (%)
*Males*						
405 - NAD(P)H	420–480	​	​	​	​	​
- Control	​	1.55 ± 0.04^a^	0.62 ± 0.03^a^	63.27 ± 0.82	3.19 ± 0.09^a^	36.73 ± 0.82
- GBH	​	1.37 ± 0.03^a,b^	0.56 ± 0.03^a,b^	65.25 ± 0.62	2.91 ± 0.07^a,b^	34.75 ± 0.62
- Gly	​	1.25 ± 0.12^b^	0.52 ± 0.03^b^	65.81 ± 3.25	2.66 ± 0.13^b^	34.19 ± 3.25
440 - FAD	500–550	​	​	​	​	​
- Control	​	1.96 ± 0.09^a^	0.83 ± 0.06	62.43 ± 1.3	3.90 ± 0.25	37.57 ± 1.3
- GBH	​	1.63 ± 0.02^b^	0.66 ± 0.03	62.11 ± 0.4	3.23 ± 0.05	37.89 ± 0.4
- Gly	​	1.71 ± 0.19^a,b^	0.76 ± 0.08	65.28 ± 1.3	3.50 ± 0.31	34.72 ± 1.3
*Females*						
405 - NAD(P)H	420–480	​	​	​	​	​
- Control	​	1.33 ± 0.04	0.50 ± 0.03	63.46 ± 0.45^a^	2.80 ± 0.10	36.54 ± 0.45^a^
- GBH	​	1.51 ± 0.11	0.58 ± 0.04	60.14 ± 1.55^a.b^	3.06 ± 0.29	39.86 ± 1.55^a,b^
- Gly	​	1.44 ± 0.03	0.57 ± 0.02	57.15 ± 2.18^b^	2.63 ± 0.09	42.85 ± 0.18^b^
440 - FAD	500–550	​	​	​	​	​
- Control	​	1.83 ± 0.06	0.77 ± 0.06^a,b^	62.28 ± 1.5	3.63 ± 0.18	37.72 ± 1.5
- GBH	​	1.86 ± 0.05	0.75 ± 0.03^a^	58.96 ± 1.2	3.33 ± 0.10	41.81 ± 1.3
- Gly	​	1.97 ± 0.05	0.89 ± 0.05^b^	58.24 ± 2.7	3.57 ± 0.26	38.20 ± 2.2

Data are presented as mean ± SEM. Different superscript letters (a, b) indicate statistically significant differences (p < 0.05) between groups of the same sex.

Regarding sex-based differences, the τ1 component of NADH was found to be lower in female Controls compared to male Controls (p < 0.05, Supplementary Figure S4A). The τ2 and τm components were similar between males and females (Supplementary Figures 4B, C, respectively). GBH- and Gly-exposed females showed a decrease in the relative fraction of free NAD(P)H (α1) and a corresponding increase in the protein-bound fraction (α2) compared to their male counterparts (p < 0.05, Supplementary Figures 4D, E, respectively). Finally, the relative fraction of protein-bound FAD (α1) in Gly-exposed females was decreased compared to Gly males (p < 0.05, Supplementary Figure 5D). There were no changes in τ1, τ2, τm or the free fraction of FAD (α2) between males and females (Supplementary Figures 5A, B, C, E).

### 3.6 Ventricular stress biomarker expression is preserved in F2 fetal hearts

**FIGURE 8:**
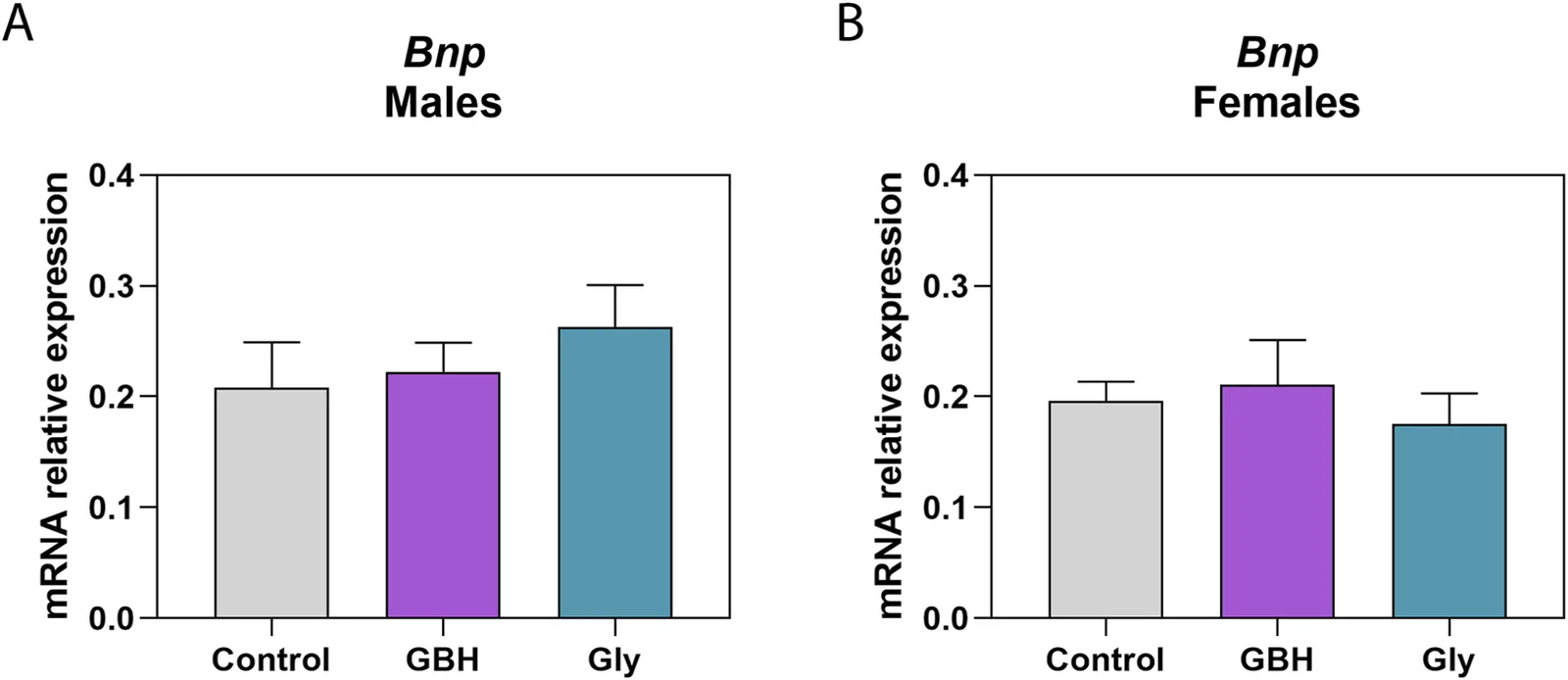
Effects of GBH or Gly exposure on relative *Bnp* mRNA expression in fetal cardiac tissue of F2 male **(A)** and females **(B)** fetuses. Relative *Bnp* mRNA expression levels are shown as mean ± SEM.

Relative *Bnp* mRNA expression did not differ among Control, GBH-, and Gly-exposed groups in either male (Figure 8A) and female fetuses (Figure 8B). No differences between sexes were detected (Supplementary Figure S6).

## 4 Discussion

This study provides the first evidence that ancestral perinatal exposure to a GBH or Gly might be associated with sex-specific alterations in fetal cardiac remodeling and metabolic regulation in the F2 generation that exhibits IUGR. Thus, the fetal heart could be proposed as a novel multigenerational target of glyphosate-induced developmental reprogramming.

In males, both GBH and Gly treatments increased relative heart weight. Since absolute heart weight remained unchanged, this increase should be interpreted in the context of the reduced fetal body weight characteristic of the IUGR phenotype. In addition, GBH induced left ventricular wall thinning and ventricular lumen dilation, consistent with a dilated phenotype of the heart (Crispi et al., 2020). GBH-exposed males also exhibited increased nuclear density without changes in cell proliferation, suggesting cellular compaction or reorganization within the ventricular wall. Conversely, Gly-exposed males showed reduced cardiomyocyte proliferation without alterations in nuclear density. Both the dilated cardiac phenotype in GBH-exposed males and the reduced cardiac proliferation in Gly-exposed males are in agreement with previous evidence from animal models of IUGR, reinforcing the biological relevance of our findings. In rabbits, IUGR fetuses exhibit a more globular cardiac shape and reduced sarcomere length (Torre et al., 2014), as well as, coronary vessel dilation as an adaptive response (Garcia-Canadilla et al., 2019). Similarly, in sheep, cardiomyocytes from IUGR fetuses were smaller, less mature, less proliferative, and reduced in number compared to Controls (Jonker et al., 2018). Moreover, clinical studies in human pregnancies complicated by IUGR have consistently documented early cardiac remodeling, often reflected in altered ventricular geometry and sphericity indices (Pérez-Cruz et al., 2015; Devi et al., 2023). Importantly, although such adaptations may initially support cardiac function and fetal growth, they may represent an early step toward long-term cardiac vulnerability (Cheong et al., 2016). In females, neither GBH nor Gly exposure altered relative heart weight. GBH reduced ventricular wall thickness without affecting lumen area, while cardiomyocyte proliferation and nuclear density remained unchanged. These findings indicate that remodeling was modest in females compared with that observed in males. The more pronounced remodeling in males, which is consistent with previous reports, highlights their greater susceptibility to developmental cardiac programming compared to females (Muralimanoharan et al., 2017; Thompson et al., 2018). Adverse intrauterine conditions are known to permanently reshape cardiac structure and metabolism as adaptive mechanisms to preserve ventricular output under restricted oxygen and nutrient supply (Crispi et al., 2020; Freedman et al., 2023). Although our findings align with this paradigm, the extent to which these sex-dependent structural changes in F2 fetal hearts represent a potential consequence of the IUGR environment or a parallel effect of ancestral exposure remains to be fully elucidated.

The structural remodeling observed in fetal hearts from F2 males, was accompanied by alterations in key molecular pathways regulating cardiomyocyte growth, inflammation, and metabolism. Although *FoxO1* expression was unchanged, altered expression of downstream targets suggests perturbation of FOXO1-mediated transcriptional networks involved in oxidative stress adaptation and metabolic regulation (Kandula et al., 2016). In males, *Dio2* expression was reduced in Gly-exposed fetuses, while *Vcam1* was increased in both GBH- and Gly-exposed groups. FOXO1–DIO2 axis plays a crucial role in regulating intracellular thyroid hormone bioavailability and cardiomyocyte maturation (Ferdous et al., 2020; Barisón et al., 2021). In line with our findings, silencing DIO2 expression in human cardiomyocytes has been associated with decreased cell proliferation and mitochondrial dysfunction (Bomer et al., 2021). To further explore local thyroid hormone signaling, we assessed *Thr-α1* gene expression, which remained unchanged, suggesting that T_3_ signaling may be preserved despite reduced DIO2. VCAM-1, in turn, is a FOXO1-regulated adhesion molecule induced by oxidative stress, inflammatory cytokines, and metabolic disturbances (Cook-Mills et al., 2011). Therefore, the increased *Vcam1* expression observed following GBH or Gly exposure could suggest a pro-inflammatory cardiac environment in male F2 fetuses. In females, gene expression remained unchanged across groups, which reinforces the idea that the absence of molecular alterations is consistent with their modest structural remodeling, and highlights their lower vulnerability to an adverse intrauterine environment.

Mitochondria play a key role in cardiac development by providing the oxidative energy necessary for growth and contractile maturation (Pohjoismäki and Goffart, 2017). During fetal life, the heart predominantly relies on glucose and lactate metabolism under relatively hypoxic conditions. Meanwhile, postnatal adaptation involves a progressive shift towards fatty acid β-oxidation and increased mitochondrial biogenesis (Drake et al., 2023; Sakamoto and Kelly, 2024). These developmental transitions ensure energetic sufficiency and define the susceptibility of the heart to intrauterine stressors. In this context, our findings reveal that exposure to GBH or Gly reshapes mitochondrial pathways in the fetal heart in a sex-dependent manner, highlighting the interplay between developmental programming and environmental insults (Gabory et al., 2011). In males, GBH exposure reduced mtDNA copy number, suggesting altered mitochondrial homeostasis consistent with IUGR-associated cardiac dysfunction (Chang et al., 2024). For instance, in a rabbit model of IUGR (Gonzalez-Tendero et al., 2013), reported a reduced mitochondrial density in fetal hearts, together with ultrastructural and transcriptional changes affecting energetic metabolism. In our work, *Pink1* expression in GBH males remained unchanged, indicating that decreased mtDNA copy number might not be driven by enhanced mitophagy. In contrast, the upregulation of *Pgc1*-α, *Nrf1*, and *Nd1* in GBH-exposed males, points to a compensatory attempt to sustain mitochondrial biogenesis and oxidative phosphorylation capacity (Iboleon-Jimenez et al., 2025). Consistent with these findings, AF-FLIM analysis revealed a decrease in FAD mean lifetime, suggesting modifications in the flavoprotein-associated microenvironment, reflecting altered engagement of oxidative metabolic pathways. In Gly-exposed males, mtDNA copy number remained unaltered, and no transcriptional modifications were observed in mitochondrial genes regulating biogenesis, oxidative phosphorylation, or mitophagy. AF-FLIM analysis shows a global reduction in NAD(P)H lifetime components (τm, τ1, and τ2) suggesting a generalized shortening of fluorescence lifetimes. This pattern might be indicative of alterations in the microenvironment of the cofactor—such as changes in pH, viscosity, or protein interactions—rather than a redistribution between free and bound states (Berezin and Achilefu, 2010). Overall, these findings show that GBH induces marked mitochondrial and metabolic reprogramming in males, whereas Gly elicits more subtle effects.

In female fetuses, mtDNA copy number, as well as *Nd1*, *Nrf1*, and *Pink1* expression, were unchanged across treatments. Nonetheless, GBH exposure led to increased expression of *Pdk4* and *Pgc1*-α, suggesting reduced glucose oxidation. Pdk4 is a downstream target of Pgc1-α, and its overexpression is associated with increased activity of mitochondrial fatty acid oxidation at the expense of glucose utilization (Pettersen et al., 2019). This metabolic reprogramming aligns with observations by Wang et al. (2013) in growth-restricted lambs, where increased glucose uptake was not efficiently metabolized due to elevated PDK4 levels.

On the other hand, Gly exposure upregulated *Pparα,* which increases fatty acid uptake and transport processes, as well as the β-oxidation enzyme *Acadl* in females*.* This was accompanied by a decrease in the relative fraction of free NAD(P)H (α1) and an increase in the protein-bound fraction (α2), suggesting a more oxidative metabolic profile. Similar metabolic reprogramming has been described by Maréchal et al. (2021), who reported that fetal cardiac tissue from female rats with IUGR exhibits increased expression of *Pparα* and β-oxidation enzymes, along with an enhanced intracellular pool of long-chain fatty acids, suggesting an early reprogramming toward lipid use for energy needs to adapt to the adverse intrauterine environment. Together, these studies suggest that such metabolic reprogramming may initially support adaptation but could reduce metabolic flexibility and increase susceptibility to cardiac dysfunction later in life (Wang et al., 2013; Maréchal et al., 2021).

Experimental and human studies have consistently demonstrated that mitochondrial organization and function are highly vulnerable to pesticide exposure. *In vitro* studies in porcine oocytes showed that Gly exposure disrupted mitochondrial distribution, reduced mtDNA copy number, and altered the expression of mitochondria-related genes, ultimately impairing meiotic maturation (Xing et al., 2022). Similarly, in a Brazilian farming population, Barbosa de Sousa et al. (2024) reported that chronic occupational exposure to pesticides was associated with significant reductions in mtDNA copy number. These findings are consistent with our results and reinforce the idea that Gly and/or GBHs may impair mitochondrial integrity and function. Such mitochondrial dysfunction could represent a key mechanistic link underlying the developmental and reproductive toxicity associated with these compounds, particularly during critical windows of fetal programming.

Early-life disturbances in mitochondrial homeostasis or energy pathways can drive the transition from compensated fetal remodeling to maladaptive hypertrophy (Wang et al., 2013). In our work, *Bnp* expression—a marker typically induced by pressure or volume overload—remained unaltered across all groups regardless of sex, and no interstitial fibrosis was detected. The stability of classical markers of pathological hypertrophy and muscle atrophy, such as *Camk2δ* and *Trim63* (Nakamura and Sadoshima, 2018), reinforces this interpretation. These findings indicate that the structural, molecular, and metabolic alterations observed correspond to early adaptive responses rather than established pathological injury.

This study has some limitations that should be acknowledged. First, no direct assessment of fetal cardiac function was performed. Although structural, molecular, and metabolic findings support the occurrence of cardiac remodeling, the functional impact of these alterations cannot be determined from the present data. Second, protein-level validation of molecular findings was not possible due to limited tissue availability, which restricts the strength of mechanistic inferences. These constraints highlight the need for future studies incorporating functional evaluations and additional morphometric and molecular markers, together with protein-level analyses, to more comprehensively characterize cardiac remodeling and its long-term implications.

Overall, our findings demonstrate that GBH exposure exerts more pronounced effects than Gly, suggesting that co-formulants are contributing to developmental toxicity (Mesnage et al., 2019; Makame et al., 2023). Moreover, these effects were also sex-dependent, with males showing greater susceptibility to females, consistent with multigenerational evidence of male-biased vulnerability to adverse cardiovascular outcomes (Muralimanoharan et al., 2017; Thompson et al., 2018; Khan et al., 2025). This pattern is also supported by our previous observations indicating a higher susceptibility of males to GBH or Gly compared to females (Almirón et al., 2026).

In summary, this study demonstrates that ancestral perinatal exposure to GBH or Gly induces early cardiac remodeling and metabolic changes in the F2 fetuses with IUGR. The alterations encompassed structural changes, modulation of cardiomyocyte growth, shifts in remodeling and metabolic gene expression, and disturbances in mitochondrial homeostasis, which varied according to treatment and sex. Notably, our results indicate that alterations correspond to early adaptive responses rather than established injury. Altogether, these findings identify the fetal heart as a multigenerational target of glyphosate herbicide-induced developmental reprograming and underscore the importance of considering both formulation and sex-specific factors into toxicological and regulatory frameworks.

## Data Availability

The raw data supporting the conclusions of this article will be made available by the authors, without undue reservation.

## Glossary

ACADL: Acyl-CoA dehydrogenase long chain
AF-FLIM: Autofluorescence lifetime images
AMPA: Aminomethylphosphonic acid
BNP: B-type natriuretic peptide
CAMKII-δ: Calcium/calmodulin-dependent protein kinase II delta
DIO2: Iodothyronine deiodinase 2
EPA: Environmental Protection Agency
FAD: Flavin adenine dinucleotide
FADH: Reduced flavin adenine dinucleotide
FOXO1: Forkhead box O1
GBH: Glyphosate-based herbicides
GD: Gestational day
Gly: Glyphosate
H&E: Hematoxylin-Eosin
IUGR: Intrauterine growth restriction
MtDNA: Mitochondrial DNA
NAD: Nicotinamide adenine dinucleotide
NAD(P)H: Reduced nicotinamide adenine dinucleotide phosphate
NADH: Reduced nicotinamide adenine dinucleotide
ND1: NADH dehydrogenase subunit 1
NRF1: Nuclear respiratory factor 1
PCR: Polymerase chain reaction
PDK4: Pyruvate dehydrogenase kinase isozyme 4
PGC1-α: Peroxisome proliferator-activated receptor gamma coactivator 1 alpha
PINK1: PTEN-induced kinase 1
PND: Postnatal day
PPARα: Peroxisome proliferator-activated receptor α
RPL19: Ribosomal protein L19
SGA: Small for gestational age
THR-α1: Thyroid hormone receptor α1
TRIM63: Tripartite motif containing 63
VCAM1: Vascular cell adhesion molecule 1
β-ACTIN: Beta-actin
