# Supplementary material for: Sex-specific cardiac remodeling and metabolic reprogramming in IUGR F2 fetuses following glyphosate or glyphosate-based herbicide exposure
Source: Front Toxicol. 2026 Aug 25;8:1870011. doi: 10.3389/ftox.2026.1870011 (PMC13549480; doi:10.3389/ftox.2026.1870011)
Supplement: Supplementary file 1 [file DataSheet1.docx]

Supplementary material

Figure 1. Effects of GBH or Gly exposure on fetal cardiac histomorphology and proliferation in F2 male and female fetuses. (A) Left ventricular wall thickness. (B) Left ventricular lumen area expressed as a percentage of total ventricular area. (C) Nuclear density in the left ventricular myocardium. (D) Cardiomyocyte proliferative activity determined by Ki-67–positive nuclei. Data are presented as mean ± SEM. Statistical differences are indicated by asterisks (*p < 0.05, **p < 0.01; ***p < 0.001).

Figure 2. Effects of GBH or Gly exposure on genes involved in cardiac remodeling in fetal cardiac tissue of F2 male and female fetuses. Relative mRNA expression levels of (A) *FoxO1*, (B) *Dio2*, (C) *Thr-α1,* (D) *Vcam1*, (E) *Camk2δ*, and (F) *Trim63*. Data are presented as mean ± SEM. Statistical significance is indicated by asterisks (*p < 0.05, **p < 0.01).

Figure 3. Effects of GBH or Gly exposure on mitochondrial regulatory pathways in fetal cardiac tissue of F2 male and female fetuses. (A) mitochondrial DNA copy number (mtDNA). Relative mRNA expression levels of (B) *Nd1*, (C) *Pink1*, (D) *Pgc1*-α, (E) *Nrf1*, (F) *Pdk4,* (G) *Pparα*, and (H) *Acadl*. Data are presented as mean ± SEM. Statistical differences are indicated by asterisks (*p < 0.05, **p < 0.01, ***p < 0.001).

Figure 4. Effects of GBH or Gly exposure on FLIM NAD(P)H parameters in fetal cardiac tissue of F2 male and female fetuses. (A) τ1, (B) τ2, (C) τm, (D) α1, and (E) α2. Data are presented as mean ± SEM. Statistical differences are indicated by asterisks (*p < 0.05).

Figure 5. Effects of GBH or Gly exposure on FLIM FAD parameters in fetal cardiac tissue of F2 male and female fetuses. (A) τ1, (B) τ2, (C) τm, (D) α1, and (E) α2. Data are presented as mean ± SEM. Statistical differences are indicated by asterisks (*p < 0.05).

Figure 6. Effects of GBH or Gly exposure on *Bnp* mRNA relative expression in fetal cardiac tissue of F2 male and female fetuses.
